# Facile Synthesis of V_2_O_5_ Hollow Spheres as Advanced Cathodes for High-Performance Lithium-Ion Batteries

**DOI:** 10.3390/ma10010077

**Published:** 2017-01-18

**Authors:** Xingyuan Zhang, Jian-Gan Wang, Huanyan Liu, Hongzhen Liu, Bingqing Wei

**Affiliations:** 1 State Key Laboratory of Solidification Processing, Center for Nano Energy Materials, School of Materials Science and Engineering, Northwestern Polytechnical University and Shaanxi Joint Lab of Graphene (NPU), Xi’an 710072, China; jackgs202@mail.nwpu.edu.cn (X.Z.); liuhuanyan@mail.nwpu.edu.cn (H.L.); liuhongzhennwpu@gmail.com (H.L.); 2 Department of Mechanical Engineering, University of Delaware, Newark, DE 19716, USA

**Keywords:** vanadium pentoxide, lithium-ion batteries, hollow spheres, cathode

## Abstract

Three-dimensional V_2_O_5_ hollow structures have been prepared through a simple synthesis strategy combining solvothermal treatment and a subsequent thermal annealing. The V_2_O_5_ materials are composed of microspheres 2–3 μm in diameter and with a distinct hollow interior. The as-synthesized V_2_O_5_ hollow microspheres, when evaluated as a cathode material for lithium-ion batteries, can deliver a specific capacity as high as 273 mAh·g^−1^ at 0.2 C. Benefiting from the hollow structures that afford fast electrolyte transport and volume accommodation, the V_2_O_5_ cathode also exhibits a superior rate capability and excellent cycling stability. The good Li-ion storage performance demonstrates the great potential of this unique V_2_O_5_ hollow material as a high-performance cathode for lithium-ion batteries.

## 1. Introduction

Rechargeable lithium-ion batteries (LIBs) are considered to be one of the most promising energy storage devices for portable devices and electric vehicles (EVs) owing to their high energy densities, environmental friendliness, and long cycle lifetime [1,2,3,4]. However, the challenges of high cost and the low capacity of the currently used cathode materials hinder their widespread applications [5,6]. In order to obtain high specific energy, considerable efforts have been devoted to developing materials with a higher specific capacity than the current commercial ones, such as LiCoO_2_ (140 mAh·g^−1^), LiMn_2_O_4_ (146 mAh·g^−1^), and LiFePO_4_ (176 mAh·g^−1^) [7,8].

Among the alternative candidates, vanadium pentoxide (V_2_O_5_) has drawn considerable attention as a cathode material for lithium-ion batteries because of its advantages of layered structure, low cost, rich abundance in nature, and a high theoretical capacity of about 294 mAh·g^−1^ (intercalation/deintercalation of two Li^+^ ions between 2.0 and 4.0 V) [9,10,11]. Nevertheless, the poor cycling stability, low electronic conductivity, and low lithium ion diffusion rate restrict its practical application [12,13,14]. Developing nanostructured materials has been demonstrated to be an effective method to address these critical issues because nanomaterials have a large surface area to provide more reaction sites and can effectively shorten the diffusion distance of lithium ions during the insertion/extraction, thereby resulting in an enhanced cycling performance and rate capability [15,16]. A variety of V_2_O_5_ nanostructures, such as nanourchins [17], nanotubes [18], nanospikes [10], nanorods [8], nanowires [19,20,21], and nanobelts [22,23,24,25], have been fabricated, and improved electrochemical performance has been achieved with these materials when used as cathodes for LIBs. In particular, hollow structures are rather favorable because the unique hollow structure can effectively buffer the volume expansion of the V_2_O_5_ cathode to improve the cycling performance [9,26,27,28]. Lou et al. reported a carbon-templating method to synthesize V_2_O_5_ hollow spheres with an excellent electrochemical performance [9]. However, the complex synthesis procedures and the expensive reactant materials limit the scale-up production. Therefore, it remains a great challenge to controllably synthesize V_2_O_5_ hollow structures through a simple and low-cost method.

In this work, we report a facile one-step solvothermal method to controllably synthesize V_2_O_5_ hollow microspheres by employing a copolymer surfactant of poly(ethylene oxide)-block-poly(propylene oxide)-block-poly(ethylene oxide) (P123) as a soft template and low-cost NH_4_VO_3_ as the vanadium source. The effects of the annealing temperature on the crystallinity, morphology, and electrochemical properties of V_2_O_5_ have been studied. The optimized V_2_O_5_ hollow microspheres, when used as LIB cathodes, exhibit a satisfactory lithium-ion storage performance.

## 2. Discussion and Results

### 2.1. Structure and Morphology of the V_2_O_5_ Microspheres

**Figure 1:**
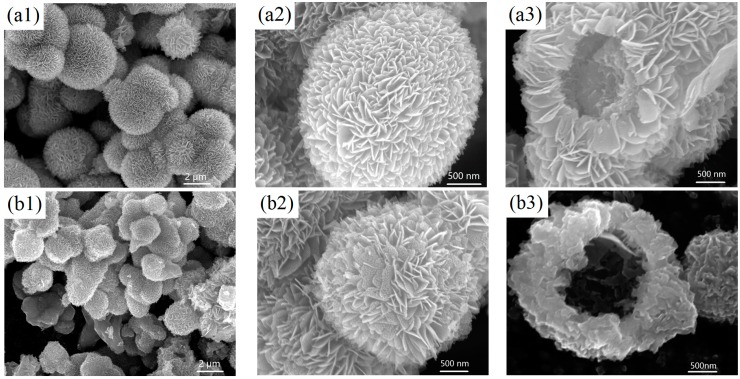

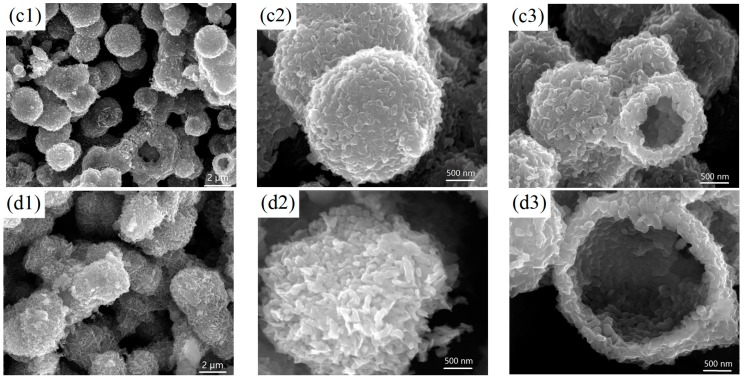
SEM images of the (**a1**–**a3**) V_2_O_5_ precursor and V_2_O_5_ microspheres annealed at 300 °C (**b1**–**b3**); 400 °C (**c1**–**c3**); and 500 °C (**d1**–**d3**).

Figure 1 shows the typical SEM images of the as-prepared samples. As shown in Figure 1a, the V_2_O_5_ precursor was composed of three-dimensional (3D) microspheres with diameters in the range of two to three microns. The magnified image in Figure 1a2 indicates that the microspheres were assembled with petal-like nanosheets. The hollow structure can be clearly observed in some broken microspheres (Figure 1a3). V_2_O_5_ was obtained by annealing the precursors, and the morphology evolution with the annealing temperature is exhibited in Figure 1b–d. The microsphere morphology was well maintained within the annealing temperature range of 300–500 °C. However, the building blocks changed with the temperature. At a low annealing temperature of 300 °C (V_2_O_5_-300), as shown in Figure 1b2, the petal-like nanosheet structure was preserved on the V_2_O_5_ microsphere surface, which was almost identical to that of the precursors. As the temperature increased to 400 °C (V_2_O_5_-400), the surface of the V_2_O_5_ hollow microspheres became constructed by interconnected nanoparticles (Figure 1c2). The nanoparticles became larger irregular structures at a higher temperature of 500 °C (V_2_O_5_-500, Figure 1d2). The morphology evolution can be attributed to the different growth rate and crystallization degree of V_2_O_5_ at different annealing temperatures [27].

**Figure 2:**
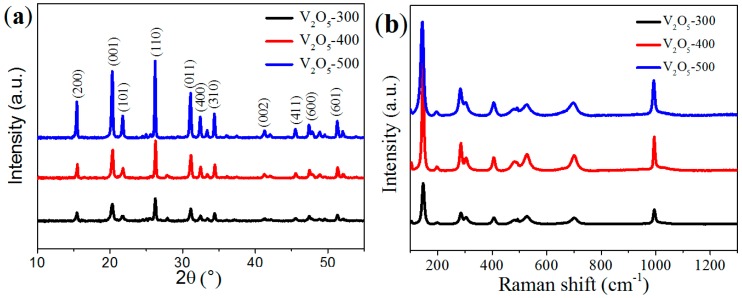
XRD patterns (**a**) and Raman spectra (**b**) of the V_2_O_5_ samples.

The crystallographic structure of the samples was characterized by X-ray powder diffraction (XRD). Figure 2a shows the XRD patterns of V_2_O_5_. Clearly, the main diffraction peaks can be well indexed to the orthorhombic phase of V_2_O_5_ (JCPDS Card No. 41-1426). In addition, the characteristic peaks showed a stronger diffraction intensity and narrower shape at an elevated annealing temperature, suggesting a higher crystallinity at a higher temperature [16,29]. Raman spectra were employed to further confirm the structure of the samples. As shown in Figure 2b, the samples shared identical Raman peaks, which are characteristic of orthorhombic V_2_O_5_ [29,30]. The typical Raman peaks V_2_O_5_ included the skeleton bent vibrations of the V-O-V bonds at 145 and 193 cm^−1^, the bending vibration of the V=O bonds at 281 and 402 cm^−1^, the bending vibrations of V_3_=O at 302 cm^−1^ and V-O-V bonds at 480 cm^−1^, and the stretching vibrations of V_3_=O bonds at 527 cm^−1^, V_2_=O bonds at 701 cm^−1^, and V=O bonds at 995 cm^−1^.

### 2.2. Electrochemical Performance

**Figure 3:**
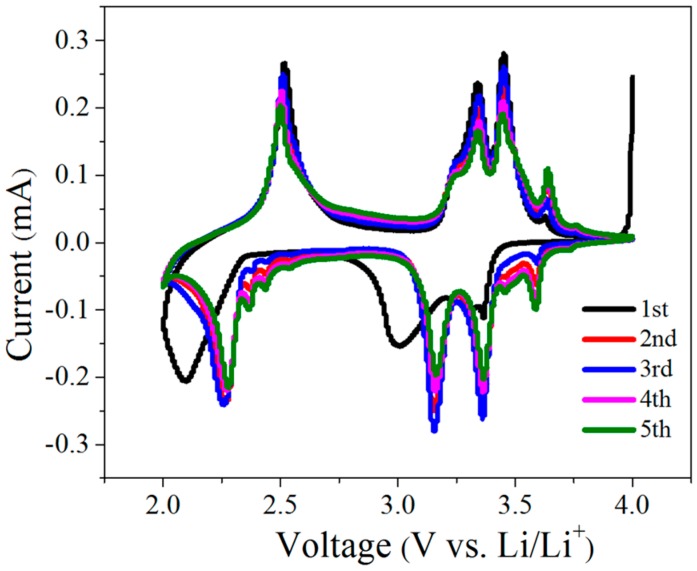
Cyclic voltammograms of V_2_O_5_-400 cathode at a scan rate of 0.1 mV·s^−1^.

The electrochemical performance of the V_2_O_5_ samples was first evaluated using the cyclic voltammogram (CV). Figure 3 shows the first five CV curves of the V_2_O_5_-400 cathode at a scan rate of 0.1 mV·s^−1^ in a potential range from 2.0 to 4.0 V. Four main pairs of redox peaks were observed at around 3.59/3.64, 3.36/3.45, 3.16/3.34, and 2.26/2.51 V, respectively, which are associated with the reversible lithium intercalation/deintercalation into/from V_2_O_5_ to form α-Li*_x_*V_2_O_5_, ε-Li*_x_*V_2_O_5_, δ-Li*_x_*V_2_O_5_, and γ-Li*_x_*V_2_O_5_, as expressed in Equations (1)–(4) [25,26,31,32]. In addition, the CV curves were well overlapped, indicating the highly reactive reversibility and good cycling stability of the cathode.

V_2_O_5_ + *x*Li^+^ + *x*e^−^ ↔ α-Li*_x_*V_2_O_5_ (*x* < 0.1) (1)

V_2_O_5_ + *x*Li^+^ + *x*e^−^ ↔ ε-Li*_x_*V_2_O_5_ (0.35 < *x* < 0.7) (2)

V_2_O_5_ + *x*Li^+^ + *x*e^−^ ↔ δ-Li*_x_*V_2_O_5_ (0.9 < *x* < 1) (3)

V_2_O_5_ + *x*Li^+^ + *x*e^−^ ↔ γ-Li*_x_*V_2_O_5_ (1 < *x* < 2) (4)

**Figure 4:**
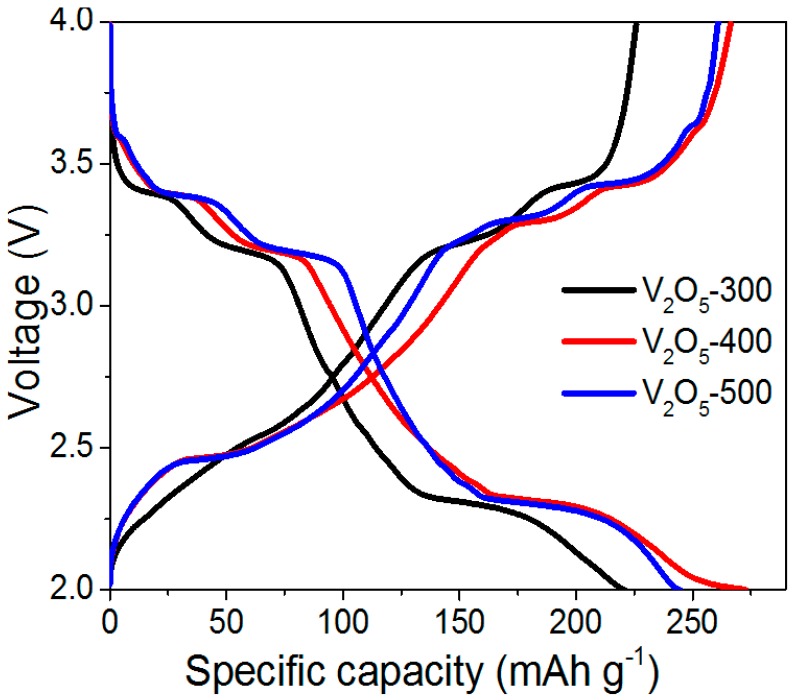
Charge-discharge curves of V_2_O_5_-300, V_2_O_5_-400, and V_2_O_5_-500 cathodes.

The electrochemical behavior of the V_2_O_5_ cathode was further investigated using the charge/discharge technique. Figure 4 exhibits the initial discharge/charge curves of V_2_O_5_-300, V_2_O_5_-400, and V_2_O_5_-500 cathodes at a current density of 0.2 C (1 C = 300 mA·g^−1^). It is observed that the three cathodes share almost identical curve shapes, indicating a similar electrochemical behavior. More specifically, four voltage plateaus at around 3.58, 3.37, 3.18, and 2.26 V in the discharge curve correspond to the multi-step lithiation of V_2_O_5_ to form α-Li*_x_*V_2_O_5_, ε-Li*_x_*V_2_O_5_, δ-Li*_x_*V_2_O_5,_ and γ-Li*_x_*V_2_O_5_, respectively, which is consistent with the CV analysis. The voltage plateaus clearly confirm the reverse-phase transformations in the discharge curve.

**Figure 5:**
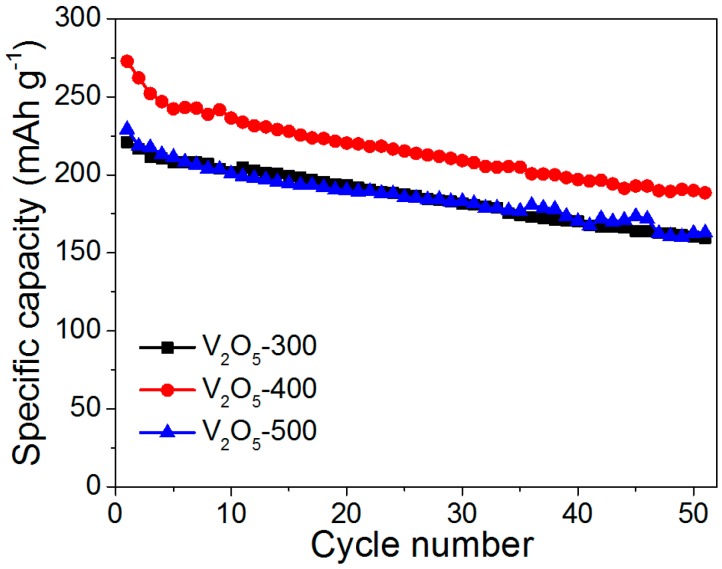
Cycling performance of V_2_O_5_-300, V_2_O_5_-400 and V_2_O_5_-500 cathodes at 0.2 C.

The cycling performance of the hollow-structured V_2_O_5_ materials was examined using the galvanostatic charge/discharge test. As shown in Figure 5, all of the V_2_O_5_ cathodes exhibited a reasonably good cycling performance over the 50 cycles at 0.2 C. It is important to note that the V_2_O_5_-400 cathode delivers an initial discharge-specific capacity of 273 mAh·g^−1^, which is close to the theoretical value (294 mAh·g^−1^). The specific capacity is also superior or comparable to most of the reported results based on differently structured V_2_O_5_ cathodes, such as the V_2_O_5_ nanorods (272 mAh·g^−1^) [8], V_2_O_5_ hollow microspheres (291 mAh·g^−1^) [9], V_2_O_5_ nanowires (278 mAh·g^−1^) [16], V_2_O_5_ nanosheets (272 mAh·g^−1^) [33] and V_2_O_5_/PEDOT/MnO_2_ (185 mAh·g^−1^) [21]. In addition, the initial specific capacity of the V_2_O_5_-400 cathode was higher than that of V_2_O_5_-300 (221 mAh·g^−1^) and V_2_O_5_-500 (229 mAh·g^−1^), indicating that the annealing temperature exerts a considerable influence on the electrochemical performance. Moreover, the V_2_O_5_-400 cathode retains a specific capacity of about 189 mAh·g^−1^ after 50 cycles.

**Figure 6:**
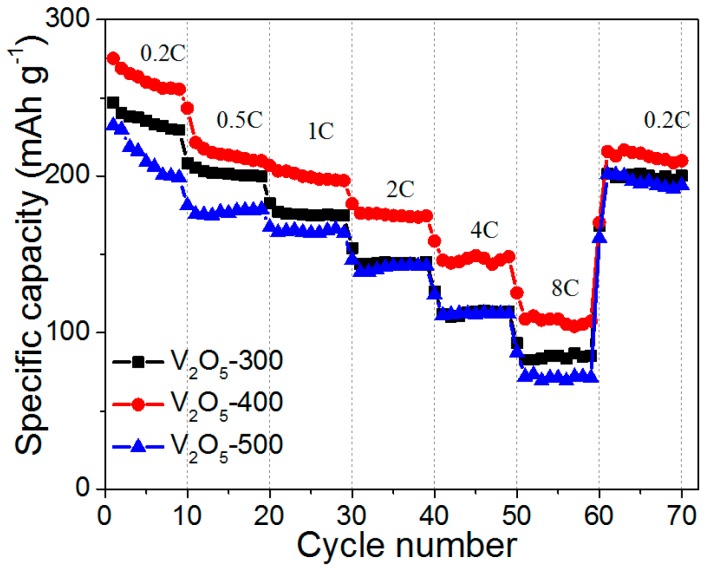
Rate performance of V_2_O_5_-300, V_2_O_5_-400 and V_2_O_5_-500 cathodes.

In order to further demonstrate the performance of V_2_O_5_ hollow microspheres, the rate capability of the cathodes at different current rates was evaluated, as shown in Figure 6. As the current rate increased from 0.2 to 8 C, the V_2_O_5_-400 cathode showed the smallest capacity decrease, indicating the best rate capability among the three cathodes. The discharge capacities of V_2_O_5_-400 cathode at the current rates of 0.2, 0.5, 1, 2, 4, and 8 C were 256, 212, 198, 173, 144 and 114 mAh·g^−1^, respectively. Even after deep cycling at 8 C, the V_2_O_5_-400 cathode could recover a specific capacity of about 210 mAh·g^−1^ when the current rate was returned to 0.2 C, further suggesting the good reactive reversibility of V_2_O_5_-400.

**Figure 7:**
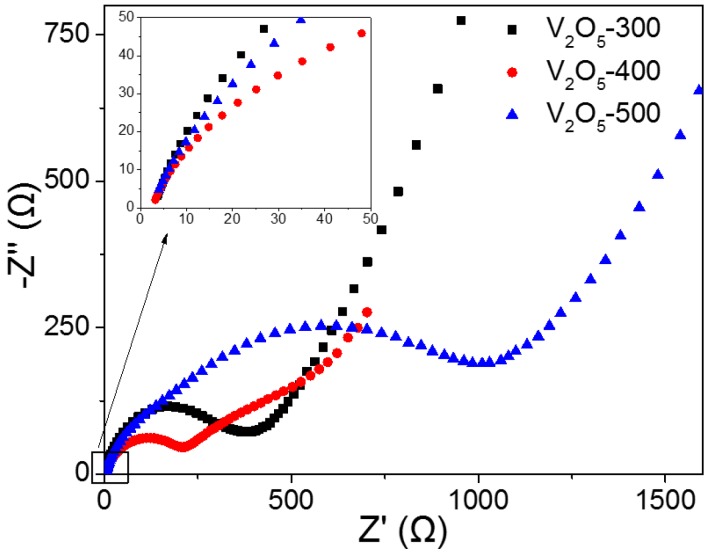
The Nyquist plots of V_2_O_5_-300, V_2_O_5_-400 and V_2_O_5_-500 cathodes (inset is the enlarged part).

Electrochemical impedance spectroscopy (EIS) analysis was performed to better understand the rate performance. Figure 7 shows the resulting Nyquist plots of the three cathodes. The Nyquist plots are composed of a depressed semicircle in the high- to medium-frequency region and an inclined line in the low-frequency region [33,34]. The *x*-intercept on the Z’ axis at the very high frequency corresponds to the bulk resistance of the electrode, and this is identical for the three cathodes (~4 Ω), as shown in the inset. The diameter of the semicircle in the high- to medium-frequency region represents the charge-transfer resistance (*R*_ct_). The inclined line in the low-frequency region is related to the Li-ion diffusion in the electrode materials. The EIS spectra of the cathodes were obtained in the same frequency range of 10 mHz–100 kHz. It is noted that the V_2_O_5_-400 cathode showed much smaller impedance in the low-frequency range, leading to the impedance of no more than 600 Ohms. The *R*_ct_ of V_2_O_5_-400 was estimated to be about 250 Ω, which is much lower than that of V_2_O_5_-300 (450 Ω) and V_2_O_5_-500 (1250 Ω). The reduced *R*_ct_ could afford fast reaction kinetics for the Li-ion intercalation-deintercalation in the V_2_O_5_-400 cathode, thereby rendering an excellent rate capability.

The good electrochemical performance of the V_2_O_5_-400 cathode can be ascribed to the unique hollow structure of the microspheres [9,29]. First, the nanoscale thickness of the shells can shorten the ion/electron transport distance for rapid charge transfer reactions; Second, the hollow space within the microspheres facilitates adequate electrolyte penetration and enlarges the effective contact area of the electrode/electrolyte interfaces; Third, the hollow structure could accommodate the volume change of V_2_O_5_ during the electrochemical processes, thus ensuring the good structural integrity of the electrode. In addition, the superior performance of V_2_O_5_-400 cathode can be ascribed to the moderate annealing temperature (400 °C) that renders high crystallinity of bulk materials and a uniform nanoparticle-assembled shell structure.

## 3. Materials and Methods

Materials Synthesis: V_2_O_5_ precursor microspheres were prepared by a solvothermal method. In a typical synthesis procedure, NH_4_VO_3_ (0.9 g) and poly(ethylene oxide)-block-poly(propylene oxide)-blockpoly(ethylene oxide) copolymer (P123) (1.5 g, *Mw* = 5800) and 6 mL of 2 mol·L^−1^ HCl were first dissolved in 90 mL of absolute ethanol under stirring. The clear mixture solution was then transferred to a Teflon-lined autoclave and solvothermally treated at 200 °C for 24 h. The precipitates were filtered and washed thoroughly with distilled water. Subsequently, the as-collected V_2_O_5_ precursor microspheres were annealed at 300, 400, and 500 °C for 2 h in air to obtain the final V_2_O_5_-300, V_2_O_5_-400, and V_2_O_5_-500 samples, respectively.

Materials Characterization: the phase structure information was obtained by X-ray diffraction (XRD, X’Pert Pro MPD, Philips, Almelo, The Netherlands) with Cu-kα radiation (1.5418 Å). The Raman spectrum was collected by Renishaw Invia RM200 (London, UK) in the spectral range of 90–1200 cm^−1^. The morphology of the samples was observed by a field emission scanning electron microscopy (FE-SEM, NanoSEM 450, FEI, Portland, OR, USA).

Electrochemical Measurements: the working electrodes were prepared by mixing active materials, carbon black, and PVDF (in a weight ratio of 70:20:10). The mixture slurry was uniformly pasted on the Al foil with a blade. The slurry-coated Al foil was dried at 120 °C in a vacuum oven overnight, followed by punching into circular electrodes with a diameter of 12 mm. The thickness of the cathode without Al foil is about 35 μm, which is more thicker than most of V_2_O_5_-based cathodes. Electrochemical measurements were carried out using coin-type cells (CR2032). Lithium plates were used as the counter electrode, and a 1 M solution of LiPF_6_ in ethylene carbon (EC)/dimethyl carbonate (DMC)/diethyl carbonate (1:1:1 *w*/*w*/*w*) was used as the electrolyte. Cyclic voltammetry (CV) and electrochemical impedance spectroscopy (EIS) were tested with a Solartron electrochemical workstation (1260 + 1287, Bognor Regis, UK). Galvanostatic charge/discharge cycling test was conducted using a Land Battery Testing system (Land, Wuhan, China). The specific capacities are calculated based on the mass of the active material.

## 4. Conclusions

In summary, V_2_O_5_ hollow microspheres were solvothermally synthesized by using P123 surfactant as the soft template and NH_4_VO_3_ as the low-cost vanadium source. The hollow-structured V_2_O_5_ can serve as an excellent cathode material for lithium-ion batteries, and it delivers a specific capacity as high as 273 mAh·g^−1^, a high-rate capacity of 114 mAh·g^−1^ at 8 C and good cycling stability. The superior electrochemical properties can be attributed to the unique hollow structure that promotes the electronic/ionic transport and accommodates the structural change. The present study also demonstrates that V_2_O_5_ hollow microspheres can be a promising alternative cathode material for next-generation lithium-ion batteries.
